# Strategies used for the COVID-OUT decentralized trial of outpatient treatment of SARS-CoV-2

**DOI:** 10.1017/cts.2023.668

**Published:** 2023-11-07

**Authors:** Nandini Avula, Dustin Kakach, Christopher J. Tignanelli, David M. Liebovitz, Jacinda M. Nicklas, Kenneth Cohen, Michael A. Puskarich, Hrishikesh K. Belani, John B. Buse, Nichole R. Klatt, Blake Anderson, Amy B. Karger, Katrina M. Hartman, Barkha Patel, Sarah L. Fenno, Neha V. Reddy, Spencer M. Erickson, David R. Boulware, Thomas A. Murray, Carolyn T. Bramante, COVID-OUT Trial Team

**Affiliations:** 1 Department of Medicine, Medical School, University of Minnesota, Minneapolis, MN, USA; 2 Investigational Drug Service, Fairview Health Services, University of Minnesota Medical Center, Minneapolis, MN, USA; 3 Department of Surgery, Medical School, University of Minnesota, Minneapolis, MN, USA; 4 Department of Medicine, Northwestern University Feinberg School of Medicine, Chicago, IL, USA; 5 Department of Medicine, School of Medicine, University of Colorado-Anschutz Medical Campus, Aurora, CO, USA; 6 UnitedHealth Group, Optum Health, Minnetonka, MN, USA; 7 Department of Emergency Medicine, School of Medicine, University of Minnesota, Minneapolis, MN, USA; 8 Department of Emergency Medicine, Hennepin County Medical Center, Minneapolis, MN, USA; 9 Department of Medicine, Olive View - University of California, Los Angeles, CA, USA; 10 Department of Medicine, School of Medicine, University of North Carolina, Chapel Hill, NC, USA; 11 Atlanta Veterans Affairs Medical Center and the Department of Medicine, Emory University School of Medicine, Atlanta, GA, USA; 12 Department of Laboratory Medicine and Pathology, Medical School, University of Minnesota, Minneapolis, MN, USA; 13 Division of Biostatistics, School of Public Health, University of Minnesota, Minneapolis, MN, USA

**Keywords:** Decentralized clinical trial, trial design, covid, remote research, randomized controlled trial

## Abstract

The COVID-19 pandemic accelerated the development of decentralized clinical trials (DCT). DCT’s are an important and pragmatic method for assessing health outcomes yet comprise only a minority of clinical trials, and few published methodologies exist. In this report, we detail the operational components of COVID-OUT, a decentralized, multicenter, quadruple-blinded, randomized trial that rapidly delivered study drugs nation-wide. The trial examined three medications (metformin, ivermectin, and fluvoxamine) as outpatient treatment of SARS-CoV-2 for their effectiveness in preventing severe or long COVID-19. Decentralized strategies included HIPAA-compliant electronic screening and consenting, prepacking investigational product to accelerate delivery after randomization, and remotely confirming participant-reported outcomes. Of the 1417 individuals with the intention-to-treat sample, the remote nature of the study caused an additional 94 participants to not take any doses of study drug. Therefore, 1323 participants were in the modified intention-to-treat sample, which was the a priori primary study sample. Only 1.4% of participants were lost to follow-up. Decentralized strategies facilitated the successful completion of the COVID-OUT trial without any in-person contact by expediting intervention delivery, expanding trial access geographically, limiting contagion exposure, and making it easy for participants to complete follow-up visits. Remotely completed consent and follow-up facilitated enrollment.

## Introduction

The severe acute respiratory syndrome coronavirus 2 (SARS-COV-2) pandemic necessitated urgent identification of effective treatments. The need to keep participants and researchers safe made conducting clinical trials challenging. In April 2020, approximately 1,000 organizations reported delays or disruptions of existing clinical trials [1]. Congruently, there was an 80% reduction in new research enrollments per site compared to 2019 [2]. Pandemic-related operational restrictions required adaptation of traditional face-to-face clinical trial methods, accelerating the development of decentralized clinical trials (DCTs). In DCTs, research interventions are remotely delivered, without requiring participants to travel to traditional in-person research sites.

The COVID-OUT trial is an example of a decentralized, quadruple-blinded, placebo-controlled, randomized controlled trial that allowed for rapid delivery of study materials to participants nationwide. The trial examined three medications (metformin, ivermectin, and fluvoxamine) as outpatient treatment for preventing progression to severe COVID-19 or long COVID in non-hospitalized adults with documented early infection. In this trial, the loss to follow-up was minimal. Metformin demonstrated meaningful clinical outcomes, and ivermectin and fluvoxamine did not [3,4]. COVID-OUT provides evidence that decentralized clinical trials are an important strategy for examining health outcomes and advancing clinical knowledge in pragmatic ways.

**Figure 1.**
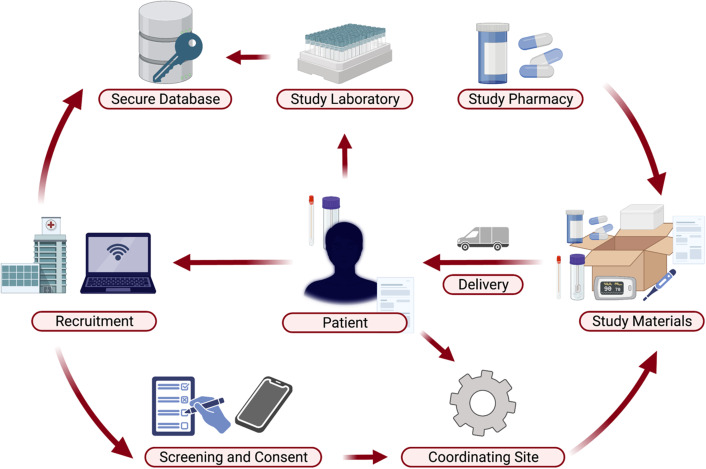
Decentralized operational elements of the COVID-OUT trial. Created with BioRender.com.

In the COVID-OUT trial, decentralized strategies facilitated coordination among six institutions, expedited medication initiation, allowed collection of multiple data points without on-site interaction, and expanded the geographic radius from which participants could enroll. Made possible by multiple streams of coordination by the lead site, the COVID-OUT trial incorporated electronic screening and consenting platforms, overnight or same-day courier services for medication delivery, and virtual follow-up by research coordinators (Table 1). We believed participants could initiate study medications, take temperature and oxygen saturation measurements, self-collect anterior nasal swabs, blood and stool samples, and self-report COVID-19 symptoms with the appropriate materials and aid from research coordinators. This premise informed the development of the decentralized design of the COVID-OUT study that ultimately provided accurate, swift, and critical clinical information (Fig. 1).

**Table 1.** Overview of adaptations made for decentralized delivery of the COVID-OUT clinical trial

Operational Element	Decentralized Adaptation
Recruitment	Clinician phone referrals
	Nationwide social media advertisements
	Emails and phone call inquiries
	Study website inquiries
Screening	Positive SARS-CoV-2 within 3 days
	Virtual self-screening form or phone call with research coordinator
Consenting	HIPAA-compliant eConsent document through REDCap
	Consent phone conversation with a research coordinator
Collecting biospecimens	Study material packaging at coordinating site
	For packing materials, could leverage volunteers who did not have to be on the IRB
Dispensing Study Drugs	Prepackaged medications into individually labeled 2-week pillboxes by study pharmacy team
	Randomization by distributing site (most often coordinating site)
Distributing Study Drug to participants	Daily study team or courier delivery from the research building where study kits were packed to the airport FedEx by 8:15pm
	Overnight or same-day shipping (FedEx overnight, FedEx Sameday) or local courier to patient residence
	This was a highly coordinated aspect of the trial, which allowed the mean time from consent to first study drug dose to be < 1 day.
Collecting Specimens and Data	Mailed paper symptom log Regular follow-up by research coordinators via secure web-based platforms and text message
	Mailed instructions for biologic specimen collection and available video assistance with research coordinators
	Return packaging for biologic specimens and symptom logs

As evidenced by multiple recent COVID-19 clinical trials [5–7], virtual platforms for recruitment, intervention delivery, and data collection are already being applied broadly. However, DCTs remain the minority, and few methodologies of large-scale studies exist in the literature. In this paper, we detail the operational elements of the COVID-OUT study, sharing the efficacy and limitations of conducting a DCT.

## Materials and Methods

### Design

This trial was a phase 3, randomized, placebo-controlled trial that employed a 2-by-3 factorial design of parallel, distinct treatments. Because groups 1 and 2 had two active drugs, all participants received 2 types of pills to maintain the blind (Table 2).

**Table 2.** Overview of the 6 randomization arms

	Metformin	Placebo	
Fluvoxamine	1: Met + Fluvoxamine	4: Placebo + Fluvoxamine	Metformin Trial: 1 + 2 + 3 vs 4 + 5 + 6
Ivermectin	2: Met + Ivermectin	5: Placebo + Ivermectin	Fluvoxamine Trial: 1 + 4 vs 3 + 6
Placebo	3: Met + Placebo	6: Placebo + Placebo	Ivermectin trial: 2 + 5 vs 3 + 6

Enrollment in the trial started on December 30, 2020, and ended on January 28, 2022. Primary end point follow-up concluded on February 14th, 2022. Six institutions in the United States enrolled participants, one serving as lead site coordinating study procedures and medications. The protocol was approved by a central Institutional Review Board and published [4].

### Inclusion Criteria

Participants had to submit documentation of a positive SARS-CoV-2 test within 3 days of randomization, affirm they had no known history of SARS-CoV-2 infection, be between the ages of 30 and 85 years, and have a body mass index in the overweight or obese categories based on self-reported height and weight. Metformin has been shown to be safe during pregnancy. Although fluvoxamine and ivermectin are not strictly contraindicated in pregnancy, fewer randomized trials have studied these effects. For this reason, pregnant patients were included in the metformin placebo arm [8,9]. Full inclusion and exclusion criteria are as published [4].

### Recruitment

Recruitment approaches were similar across sites. Patients scheduled for SARS-CoV-2 testing at participating sites were informed of the trial before testing and before receiving their results via brochures, electronic messages, or phone calls. Close contacts of patients with a positive SARS-CoV-2 test result were proactively alerted of the study. Patients who received their test results over the phone by the clinical team were notified that they may be eligible for this research study and given study team contact information. Additionally, information about the trial was sent to all local, then many regional and national, testing clinics to advertise the study to individuals receiving testing. Patients who reached out to community patient advocacy groups about enrolling in a clinical trial were also contacted about the study. Nationwide social media posts, print newspaper ads, flyers, and Google ads were posted with enrollment information. Recruitment outside of health systems eventually accounted for most enrollments, primarily through the lead site due to bandwidth at participating sites.

Research coordinators responded to patient emails, study website inquiries, and phone calls immediately. The study team used a centralized telephone number with waterfall software that routed incoming calls to research personnel that were available so that all incoming phone calls could be answered in real-time, without potential participants being directed to voicemail and then re-contacted. Answering the phone in real-time was an important way to capture individuals when they were most interested in learning more about the study.

### Screening

A secure online form through Research Electronic Data Capture (REDCap) was available to participants who elected to self-screen online by self-reported responses to eligibility questions. Most participants were screened over the phone while a research coordinator entered eligibility information into the REDCap database. Patients submitted documentation of a positive SARS-CoV-2 result through secure email or directly onto the secure REDCap screening form. Patients could be randomized before sending confirmation of a positive result, but they were encouraged to send it immediately, before randomization. Failure to provide documentation of a positive SARS-CoV-2 result meant they were ineligible. Preexisting medical conditions and home medications were recorded in the study database at baseline.

Because metformin use is cautioned with a glomerular filtration rate (GFR) < 30ml/min, serum creatinine was assessed in persons at high risk for decreased GFR: age > = 75 or a history of chronic kidney, heart, or liver failure. If serum creatinine (sCR) was not available in the electronic health record (I) within 2 weeks, blood collection was required. Patients could choose in-person collection or self-collect blood samples that were returned to the lab via overnight FedEx. Study medications could be delivered and initiated while sCr results were pending; few days of metformin carries minimal risk in an individual with a GFR < 45. If the GFR returned as < 45 ml/min, participants were not eligible and instructed to discontinue and return study drugs via prepaid materials. By being proactive, study medication could be delivered as soon as possible to the maximum number of participants. In total, 14 (1%) participants who were randomized were then withdrawn based on the above operational criteria. The study team felt this was an acceptable amount of forgone cost to allow faster delivery of study medication to all potential participants.

### Consent

REDCap made it possible to complete eConsent that was compliant with FDA 21 CFR Part 11 for electronic signature capture. The eConsent document with the full consent text was sent to potential participants through REDCap and trained study staff assessed consent comprehension questions over the phone. Individuals could also self-consent online if they successfully read through short sections of the consent document followed by comprehension questions. Potential participants using the self-consent could also elect to have a consent conversation with a research coordinator over the phone at any time.

### Enrollment and Randomization

After consent, a HIPAA-compliant email was immediately sent to onsite study personnel with participant weight, address, age, and pregnancy status. Weight and pregnancy/ lactational status were necessary because ivermectin was weight-based, and the ivermectin and fluvoxamine arms were not open for pregnant individuals. The research team assured that at least one research coordinator, and not more than two to limit COVID exposure, was on-site daily to then randomize participants to a treatment arm using a preprogramed randomization app.

### Dispensing and Distribution of Medications

Because two study arms included two active medications, all participants received two types of pills to maintain blinding. All participants received metformin or metformin placebo, and then a subset received ivermectin or fluvoxamine or their exact matching placebos. Ivermectin was weight-based, so each dose required between 2 and 4 pills. To ensure that participants took the right number of pills, study medications were dispensed by the pharmacy team into 2-week pillboxes. Distinct blinded study packs were created for pregnant and lactating women containing only metformin or placebo.

Due to operating hours, a new participant needed to be randomized before 3:30 pm for the investigational pharmacy to individually dispense the participant’s study drug the same day. However, most enrollments were after 3:30 pm. To ensure that the study medication was sent to the patient on the day of consent, the study pillboxes were pre-packed by the pharmacy with individual packet ID’s. The pillboxes were stored in the research team’s dedicated, locked office area. When a research coordinator randomized a new patient, the randomization app assigned one of the individual packet ID’s to the new participant ID (PID). The research coordinator then entered the individual packet ID with the PID, participant name, and DOB, into the paper drug accountability log.

Research coordinators then put the study drug into study kits with the other materials (symptom log, oximeter, nasal swab, and stool collection material) and applied a FedEx label addressed to the participant’s home. Every evening a study team member took the kits for participants enrolled that day to the FedEx at the airport. The airport FedEx was open until 8:15 pm each night, and everything that arrived there by 8:15pm was guaranteed to be delivered the next day via FedEx overnight shipping. To alleviate the burden on study team members so they could focus on follow-up with participants, a certified medical courier was contracted to arrive at the research building daily at 7:45 pm and make a direct route to the airport FedEx. The study team received an email alert from FedEx when each study kit was scanned at FedEx.

Thus, prepacking and distribution by the study team meant that every participant enrolled before 7:45 pm, rather than 3:30 pm, would receive study drugs the next day, nationwide. Participants who enrolled within a 4-hour radius of the 6 participating institutions received study drugs the same day via same-day courier. The independent external study monitor conducted both remote and in-person monitoring to review the paper drug accountability log.

### Study Materials

The materials were packaged into cardboard boxes containing a daily symptom log, ClinCard [10] for compensation, pulse oximeter, thermometer, and approved shipping materials for return samples. Instant cold packs and mini foam coolers were provided for temperature stability of the biological samples. If a GFR was required at the time of screening, then materials for collecting blood via finger prick were included. Assembling the materials for nasal swab collection was time-intensive but did not involve protected health information, so volunteers not on the formal study team could help assemble materials. The study team members placed labels with the participant’s ID and a barcode unique to the participant on the symptom log, nasal swabs, and stool collection kits once randomized.

### Data and Lab Collection

Each study kit contained a paper symptom log with prompts for participants to record their COVID-related symptoms on a 4-point scale [11]. Participants also reported medication adherence, temperature, and oxygen saturation using the study-provided home oxygen monitor for 14 days. Research coordinators contacted patients at specified follow-up points to assess clinical progression of COVID-19.

The symptom log included pictorial instructions for nasal and blood specimen collection. Patients who collected SARS-CoV-2 PCR nasal swabs on Days 1, 5, and 10 or self-collected a finger prick GFR blood test could request help from research coordinators over the phone or by secure video. For optional stool samples, each microbiome self-collection kit included a paper copy of detailed collection procedures (Table 3). Study coordinators arranged for FedEx to receive the biologic specimens and return them to the lead site lab via overnight delivery (or same day with local courier). Participants mailed back symptom logs in pre-addressed and prepaid United States Postal Service envelopes.

**Table 3.** Number of biospecimens submitted by participants

Number of samples submitted	Day 1	Day 5	Day 10
Blood samples	31	33	23
Nasal swab samples	945	871	775
Stool samples	221	142	114

### Follow-up Procedures

Research coordinators contacted participants at various time points using the participant’s preferred contact method (phone, email, or secure text message). On day 1, research coordinators confirmed that medications were received and started. They also ensured that any labs (GFR or nasal swab) were properly collected and return shipping arranged. On day 2, side effects of the drugs were assessed using the PROMIS Gastrointestinal Symptoms survey and recorded in REDCap [12]. Other side effects, adverse events, infection progression, and symptom logs were also monitored and noted. If GFR returned < 45 ml/min, study medication return was arranged. Research coordinators assessed self-reported concomitant medication use, study drug discontinuation, and clinical progression to severe COVID-19 on Days 5 and 10.

On day 14, research coordinators again assessed concomitant medication use and clinical progression, study drug discontinuation, and symptom log return. At day 28, symptoms, clinical progression, and any additional medications used during the study duration were recorded. Participants were paid throughout the study with each milestone: enrollment; follow-up assessments at Day 1, 5, 10, 14, 28, and then long-COVID assessments. The payments were made via the ClinCard system [10], an electronic debit card system.

## Results

Of 6,609 individuals screened, 5,178 were excluded and 1,431 were randomized; 14 individuals were ineligible after randomization: they provided inaccurate screening information, could not provide proof of SARS-CoV-2 infection, or withdrew immediately after completing randomization.

### Trial Sample

Of the 1,417 in the intention-to-treat (ITT) sample, 94 participants informed study members that they took no study drug: 9 participants did not receive the study drug due to shipping failure; 8 were hospitalized before taking study drug; and 77 were no longer interested in taking study drug (consenting to study procedures) before they received the shipment. Therefore, 1,323 were in the modified intention-to-treat sample, which was the prespecified primary study sample. The difference between the ITT and mITT samples is due to the remote nature of the trial. Unlike in-person trials, when participants took the first dose on site, there was a lag time between consenting and receiving the trial intervention.

Of the 1,323 in the primary sample, the COVID-OUT study enrolled fewer black (7.6%) participants compared to the general U.S. population (about 13%). The COVID-OUT trial population was similar to the US population for percent Native American (about 2%), and under-enrolled 12.1% Latinx, compared to about 18% Latinx for the US population.

### Definition and Internal Validity of Primary Outcome Data

Eighteen participants (1.4%) were lost to follow-up for the primary outcome: severe Covid-19 within 14 days. Severe Covid-19 was defined using a binary 4-part composite outcome: (1) single oxygen reading < 94% (2) ED visit (3) Hospitalization (4) or Death. While little was known at the time about silent hypoxia due to COVID-19, one low oxygen reading does not equal severe COVID-19. Additionally, some participants reported improbable, non-physiologic variability of pulse oximetry readings (e.g. 99% going to 75% going to 98% in the same day, or some values over 100%). This may have been due to participant reporting of pulse rate instead of oxygen saturation, a falsely low reading due to the vasoconstriction of cold extremities, poor fit, skin tone, or inaccurate pulse oximetry readings. After the study began enrolling, the FDA issued a warning that home pulse oximeters can be inaccurate [13]. Using a reading of < 94% on a home oximeter (an FDA criteria for severe COVID-19) as indication of severe COVID-19 introduced random noise into the composite primary endpoint. Individuals in both treatment and control conditions were classified as having severe COVID-19 even though they did not [4,14]. The other primary composite outcome components (emergency room visit, hospitalization, or death) had much greater internal validity and each event was verified by obtaining source documentation. Verifying patient- or family-reported medical events with external documentation was important: one family-reported death did not actually occur; the participant was in prison.

We chose to focus on the primary outcomes within 14 days of study drug initiation with 28-day outcomes as secondary endpoints. Pharmaceutical industry-sponsored trials focused on 28-day outcomes. In the COVID-OUT trial, there was a clinically meaningful reduction in hospitalizations by 14 days, but the confidence interval included 1.0. Through 28-day follow-up, the confidence interval did not include 1.0 [4]. While secondary endpoints may be used to influence guideline committees [14,15], this experience will inform our future primary outcomes as longer follow-up is statistically more powerful.

## Discussion

The SARS-CoV-2 pandemic limited in-person clinical trial activities, prompting investigators to leverage virtual clinical trial methods. The surge in DCT development during the COVID-19 pandemic represents an inflection point that could transform the future of clinical research. Decentralized methods can enhance efficiency of intervention assessment and expand clinical trial access for patients who live far from medical centers, thereby improving generalizability of clinical trials despite infectious pandemics.

Although our DCT strategies were not compared to in-person methods, our experience suggests that conducting the COVID-OUT trial remotely conferred several advantages. Most notably, the decentralized trial design adhered to current COVID-19 safety guidelines [16]. Patients who tested positive for COVID-19, a highly contagious virus, remained quarantined while participating in the trial. DCTs could be applied to other infectious agents to limit contagion exposure and decrease burden of research participation. The decentralized nature also likely improved enrollment because many individuals may not have participated in an in-person trial while feeling acutely ill.

Expedited medication delivery was a critical component of this trial. Delivering study medications within 24 hours of enrollment improves statistical power for antiviral medications because earlier initiation is associated with larger effect sizes [17]. Additionally, only 1.4% of participants were lost to follow-up due to consistent follow-up and coordination. With proper assistance, the right tools, and monitoring by research coordinators, the COVID-OUT trial illustrates that participants can collect clinically meaningful data without on-site visits.

### Lessons Learned and Limitations

We now know that a one-time low reading on a home oximeter does not equal severe COVID-19. The FDA-identified inaccuracies of prescription home oxygen monitoring devices, combined with other potential issues likely influenced the accuracy of hypoxemia assessment. This had an overwhelming effect on the primary outcome because reported hypoxia was the most frequently occurring component of the binary, 4-part composite outcome. Real-time data entry into REDCap would have alerted the study team to spurious or low values, allowing real-time verification with the patient over the phone. As an alternative, when remotely collecting vital sign data, the addition of a related symptom may improve accuracy. For example, a low oxygen reading plus shortness of breath may better assess pulmonary involvement of COVID-19. Medication adherence was also monitored using self-reported symptom log data. Objective measures such as return of an empty pill pack or video drug intake observation may have informed researchers of true medication adherence.

Initially, most participants were enrolled in participating health systems and thus their EHR could be reviewed. However, the EHR may not contain all follow-up events or new medications, so asking the participant is always preferred. While all participants consented to EHR access, home and new medications started during the trial were not confirmed in the EHR as obtaining records from clinics outside of participating systems would have been unreasonable.

While paper symptom logs are part-11 compliant, paper symptom diaries created a significant data entry burden. The delay between receiving diaries and entering data prevented real-time quality control with participants who may have misread home oximeters, recording their pulse. Non-return of symptom diaries created missing symptom data. A subset of participants may have preferred paper symptom diaries but having an alternative direct electronic entry option would have been more appropriate.

Early in the study, participants would forget to label their nasal swabs and stool samples with their participant-specific adhesive sticker. The burden of determining missing identification numbers fell upon lab personnel and study staff. In response to this challenge, research coordinators placed adhesive stickers labeled with day of collection and participant ID before shipping materials to participants. By minimizing what is required of the participant, more accurate data collection occurred.

Initial data suggest that DCTs may improve sample diversity compared to clinic-based trials by reducing the geographic and time barriers that contribute to underrepresentation of people of color in clinical research [18]. However, inequalities persist with 79% of white households having access to broadband internet compared to only 66% and 61% of African American and Hispanic households, respectively [19,20]. This digital divide may explain why the racial and ethnic demographics in the COVID-OUT and other decentralized COVID-19 clinical trials do not represent the groups most affected by the COVID-19 pandemic, highlighting the need to adapt decentralized strategies when technology access and literacy are limited [21]: potentially leveraging surveys administered over the phone, technology delivery for the duration of a DCT, community partnerships, and an open technical support line.

Geotargeting digital advertisements and partnering with clinical sites that serve diverse communities may also enhance recruitment. DCTs present a unique opportunity to meet participants within the communities they live, separate from traditional sites. Collaborating with community organizations for research strategy could strengthen the researcher-participant relationship.

DCTs come with several challenges and advantages, both of which must be weighed in the context of the trial being designed. We hope that learning the details of the COVID-OUT trial will be helpful to other researchers and add to the emerging literature on decentralized clinical trials.

## Supporting information

Avula et al. supplementary material Avula et al. supplementary material

## Supplementary material

The supplementary material for this article can be found at https://doi.org/10.1017/cts.2023.668.
