# Supplementary material for: Strategies used for the COVID-OUT decentralized trial of outpatient treatment of SARS-CoV-2
Source: J Clin Transl Sci. 2023 Nov 7;7(1):e242. doi: 10.1017/cts.2023.668 (PMC10685265; doi:10.1017/cts.2023.668)
Supplement: Avula et al. supplementary material [file S2059866123006684sup001.docx]

**Supplementary Material**

**Table of Contents**

[1](#_Table_1._COVID-OUT) [COVID-OUT Study Team](#_Table_1._COVID-OUT) 2

# **Table 1. COVID-OUT Study Team**

| **Name** | **Institute** | **Location** |
| --- | --- | --- |
| Blake Anderson | Emory | Atlanta, GA |
| Riannon C Atwater | University of Colorado | Aurora, CO |
| Nandini Avula | University of Minnesota | Minneapolis, MN |
| Kenny B Beckman | University of Minnesota | Minneapolis, MN |
| Hrishikesh K Belani | Olive View - UCLA | Sylmar, CA |
| David R Boulware | University of Minnesota | Minneapolis, MN |
| Carolyn T Bramante | University of Minnesota | Minneapolis, MN |
| Jannis Brea | Northwestern University | Chicago, IL |
| Courtney A Broedlow | University of Minnesota | Minneapolis, MN |
| John B Buse | University of North Carolina | Chapel Hill, NC |
| Paula Campora | University of Minnesota | Minneapolis, MN |
| Jill Charles | University of Minnesota | Minneapolis, MN |
| Grace Christensen | University of Minnesota | Minneapolis, MN |
| Theresa Christiansen | M Health Fairview | Minneapolis, MN |
| Ken Cohen | Optum | Minnetonka, MN |
| Bo Connelly | University of Minnesota | Minneapolis, MN |
| Srijani Datta | University of Minnesota | Minneapolis, MN |
| Nikita Deng | University of Colorado | Aurora, CO |
| Alex T Dunn | Hennepin Healthcare | Minneapolis, MN |
| Spencer M Erickson | University of Minnesota | Minneapolis, MN |
| Faith M Fairbairn | University of Minnesota | Minneapolis, MN |
| Sarah L Fenno | University of Minnesota | Minneapolis, MN |
| Daniel J Fraser | University of Minnesota | Minneapolis, MN |
| Regina D Fricton | Feinberg School of Medicine, Northwestern | Chicago, IL |
| Gwen Griffiths | University of Minnesota | Minneapolis, MN |
| Aubrey A Hagen | University of Minnesota | Minneapolis, MN |
| Katrina M Hartman | University of Minnesota | Minneapolis, MN |
| Audrey F Hendrickson | Hennepin Healthcare | Minneapolis, MN |
| Jared D Huling | University of Minnesota | Minneapolis, MN |
| Nicholas E Ingraham | University of Minnesota | Minneapolis, MN |
| Arthur C Jeng | Olive View - UCLA | Sylmar, CA |
| Darrell M Johnson | University of Minnesota | Minneapolis, MN |
| Amy B Karger | University of Minnesota | Minneapolis, MN |
| Nichole R Klatt | University of Minnesota | Minneapolis, MN |
| Erik A Kuehl | M Health Fairview | Minneapolis, MN |
| Derek D LaBar | M Health Fairview | Minneapolis, MN |
| Samuel Lee | Feinberg School of Medicine, Northwestern | Chicago, IL |
| David M Liebovitz | Feinberg School of Medicine, Northwestern | Chicago, IL |
| Sarah Lindberg | University of Minnesota | Minneapolis, MN |
| Darlette G Luke | M Health Fairview | Minneapolis, MN |
| Rosario Machicado | Olive View - UCLA | Sylmar, CA |
| Zeinab Mohamud | University of Minnesota | Minneapolis, MN |
| Thomas A Murray | University of Minnesota | Minneapolis, MN |
| Rumbidzai Ngonyama | University of Minnesota | Minneapolis, MN |
| Jacinda M Nicklas | University of Colorado | Aurora, CO |
| David J Odde | University of Minnesota | Minneapolis, MN |
| Daniela Parra | University of Minnesota | Minneapolis, MN |
| Barkha Patel | University of Minnesota | Minneapolis, MN |
| Jennifer L Proper | University of Minnesota | Minneapolis, MN |
| Matthew F Pullen | University of Minnesota | Minneapolis, MN |
| Michael A Puskarich | Hennepin Healthcare | Minneapolis, MN |
| Via Rao | University of Minnesota | Minneapolis, MN |
| Neha V Reddy | University of Minnesota | Minneapolis, MN |
| Naveen Reddy | Northwestern University | Chicago, IL |
| Katelyn J Rypka | University of Minnesota | Minneapolis, MN |
| Hanna G Saveraid | University of Minnesota | Minneapolis, MN |
| Paula Seloadji | Olive View - UCLA | Sylmar, CA |
| Arman Shahriar | University of Minnesota | Minneapolis, MN |
| Nancy Sherwood | University of Minnesota | Minneapolis, MN |
| Jamie L Siegart | University of Colorado | Aurora, CO |
| Lianne K Siegel | University of Minnesota | Minneapolis, MN |
| Lucas Simmons | University of Minnesota | Minneapolis, MN |
| Isabella Sinelli | University of Colorado | Aurora, CO |
| Palak Singh | University of Minnesota | Minneapolis, MN |
| Andrew Snyder | M Health Fairview | Minneapolis, MN |
| Maxwell T Stauffer | St. Olaf College | Northfield, MN |
| Jennifer Thompson | Vanderbilt University | Nashville, TN |
| Christopher J Tignanelli | University of Minnesota | Minneapolis, MN |
| Tannon L Tople | University of Minnesota | Minneapolis, MN |
| Walker J Tordsen | Hennepin Healthcare | Minneapolis, MN |
| Ray HB Watson | University of Minnesota | Minneapolis, MN |
| Beiqing Wu | University of Minnesota | Minneapolis, MN |
| Adnin Zaman | University of Colorado | Aurora, CO |
| Madeline R Zolik | M Health Fairview | Minneapolis, MN |
| Lena Zinkl | M Health Fairview | Minneapolis, MN |
